# Prevalence of Etiological Factors in Adult Patients With Epilepsy in Herzegovina

**DOI:** 10.7759/cureus.82184

**Published:** 2025-04-13

**Authors:** Natasa Pejanovic-Skobic, Asja Hodzic, Nikolina Pravdic, Marija Bender, Jelena Kordic

**Affiliations:** 1 Department of Neurology, University Clinical Hospital Mostar, School of Medicine, University of Mostar, Mostar, BIH; 2 Department of Neurology, University Clinical Hospital Mostar, Mostar, BIH; 3 Department of Anesthesia, Reanimation, and Intensive Care, University Clinical Hospital Mostar, Mostar, BIH

**Keywords:** adult, epilepsy, etiology, patients, prevalence

## Abstract

Objective: This study aims to assess the prevalence of etiological factors contributing to epilepsy in adult patients in Herzegovina.

Methods: The study included all patients with a diagnosis of epilepsy who were older than 18 years and were examined in the epilepsy outpatient clinic or hospitalized in the Department of Neurology, University Clinical Hospital Mostar, during the period from January 2023 to December 2023.

Results: Of the 421 patients, 217 were women and 204 were men. Structural factors were the most frequent causes, with brain tumors being the most prevalent among them. Immune factors were the rarest causes of epilepsy. It was found that the biggest difference between women and men existed in the group of patients with brain trauma (p < 0.001) as the cause of epilepsy. A statistically significant difference in frequency was found when it came to structural epilepsies compared to other causes of epilepsy (p < 0.001). Within the group of structural factors, a statistically significant difference was observed in patients with brain tumors (p < 0.001) and stroke (p < 0.001). Men were slightly older than women, but there was no statistically significant difference. Focal epilepsy was the most common type of epilepsy (47.1% of men and 51.2% of women), and subjects with focal epilepsy were statistically significantly older than subjects with generalized epilepsy (p < 0.001).

Conclusion: This retrospective study provided the first insights into the prevalence of etiological factors in our region. Among the studied sample, structural factors were the most common cause of epilepsy, while immunological factors were the least common. Within the structural etiology category, tumors were the most frequent factor, whereas perinatal insults were the rarest.

## Introduction

Epilepsy is one of the most common neurological diseases, affecting approximately 70 million people, and its incidence varies by age, sex, race, type of epilepsy, and socioeconomic status [1]. Approximately 1% of the population suffers from epilepsy, and about one-third of patients have refractory epilepsy, i.e., seizures that are not controlled with two or more appropriately selected antiepileptic drugs or other therapies. The International League Against Epilepsy (ILAE) accepted the recommendations of a working group proposing that epilepsy should be considered as a brain disease defined by any of the following conditions: the occurrence of at least two unprovoked (or reflex) epileptic seizures more than 24 hours apart, the occurrence of one unprovoked (or reflex) seizure with a probability of further seizures of at least 60% after two unprovoked seizures, occurring over the next 10 years, or a diagnosis of epileptic syndrome [2].

In 2017, the ILAE introduced three levels of diagnosis: seizure type, epilepsy type, and epileptic syndrome [3]. It describes three classification levels starting with seizure type, assuming the patient has epileptic seizures as defined by the 2017 ILAE seizure classification update [4]. The updated classification emphasizes the importance of considering the underlying cause at each stage of diagnosis, as it frequently has significant implications for treatment. ILAE defined six etiologic categories for epilepsy: structural, genetic, metabolic, infectious, immunological, and unknown. They are not hierarchical, and epilepsy in patients can be classified into more than one etiological category.

A structural etiology involves detectable abnormalities on structural neuroimaging, where the combination of electroclinical evaluation and imaging results supports a well-founded conclusion that the identified abnormality is the probable cause of the patient's seizures [3]. The root cause of a structural abnormality may be genetic, acquired, or a combination of both. Hippocampal sclerosis is marked by neuronal cell loss in specific hippocampal regions and presents a common finding in mesial temporal lobe seizures [4]. Brain tumors, such as gangliogliomas and dysembryoplastic neuroepithelial tumors, are a frequent cause of seizures in the pediatric population. In early childhood, drug-resistant epilepsy is often associated with malformations of cortical development, with focal cortical dysplasia (FCD) being the most prevalent form linked to epilepsy. In adults, stroke is one of the most prevalent causes of epilepsy. Notably, epilepsy can be diagnosed following a single late post-stroke seizure, which occurs more than seven days after the stroke, due to the high risk of recurrence exceeding 60% within the subsequent decade [5]. Vascular malformations and traumatic brain injuries are additional structural factors that may contribute to the development of epilepsy.

Epilepsy is classified as having a genetic origin if a known or suspected disease-causing gene variant or copy number variation is present, where seizures represent a common clinical manifestation [3]. It is estimated that around 30% of all epilepsy cases have a genetic origin [6]. They encompass both monogenic forms, which may involve inherited or de novo pathogenic variants, and complex forms, which are polygenic and may be influenced by environmental factors. Over 500 genes associated with epilepsy have been identified [7]. Acknowledging the crucial impact of de novo pathogenic variants, the ILAE classification no longer requires proof of genetic inheritance. Clinically, genetic epilepsies can be categorized into generalized genetic epilepsies, focal genetic epilepsies, and developmental and epileptic encephalopathies (DEE) [3]. Genetically generalized epilepsies typically emerge during childhood or adolescence and are marked by generalized seizures impacting both hemispheres of the brain. Conditions such as juvenile myoclonic epilepsy and childhood absence epilepsy fall under this category, with deletions on chromosomes 15q13.3, 16p13.11, and 15q11.2 being identified as associated genetic factors [8,9]. Some examples of focal generalized epilepsies include familial mesial temporal lobe epilepsy (FMTLE), autosomal dominant lateral temporal epilepsy (ADLTE), and autosomal dominant nocturnal frontal lobe epilepsy (ADNFLE). Epileptic encephalopathies are severe, early-onset disorders characterized by treatment-resistant seizures, developmental delay, or regression due to persistent epileptic activity, and generally poor outcomes. This category includes various conditions caused by mutations in genes encoding ion channels, such as KCNQ2 in benign familial neonatal seizures, SCN2A in benign familial infantile epilepsy, and SCN1A in Dravet syndrome [10-12].

Infectious etiology in the central nervous system can lead to both acute symptomatic (provoked) seizures, which are closely linked to the timing of the initial infection, and epilepsy. Infections can be prion, viral, bacterial, fungal, and parasitic [3]. In this context, seizures are triggered by brain tissue damage, toxin production by the infectious agent, or the onset of inflammation. Infections of the central nervous system are especially common in developing countries, and the occurrence of seizures poses a significant risk factor for mortality in this population of patients [13]. Some examples include neurocysticercosis, the most common parasitic brain infection worldwide [14], tuberculosis, toxoplasmosis, and malaria [3].

Metabolic causes of seizures and epilepsy can be either acquired or genetic (inborn). Acquired metabolic causes often result from organ failure, nutritional deficiencies, systemic autoimmune conditions, or the influence of exogenous drugs and toxins [15]. In contrast, inborn errors of metabolism typically manifest in early childhood, with over 200 genetic metabolic disorders associated with epilepsy. Seizures in these cases can be attributed to a variety of mechanisms, including disruptions in brain metabolism, deficiencies in vitamins or cofactors, the accumulation of toxins or abnormal storage materials, disturbances in neurotransmitter systems, or the presence of related malformations of cortical development [16,17]. Specific examples of these conditions include mutations in the SLC2A1 gene, which encodes the glucose transporter GLUT1, as well as metabolic disorders such as mitochondrial encephalomyopathy, lactic acidosis, and stroke-like episodes (MELAS) and myoclonic epilepsy with ragged red fibers (MERRF) syndromes.

For epilepsy to be directly caused by an immune disorder with seizures as a primary symptom, there must be evidence of autoimmune-induced inflammation in the central nervous system (CNS). It is crucial to differentiate between acute provoked seizures caused by autoimmune encephalitis and chronic unprovoked seizures resulting from autoimmune-associated epilepsy. Chronic "unprovoked" seizures associated with autoimmune epilepsy occur in autoimmune encephalitis patients who have autoantibodies against intracellular neural antigens and those with Rasmussen's encephalitis. In these cases, T cells are believed to trigger epileptogenic effects through immune inflammation and significant structural damage. These patients show a persistent predisposition to seizures even after the "acute phase" of encephalitis, thereby meeting the criteria for epilepsy [18]. The diagnosis of autoimmune encephalitides is rapidly increasing, driven by improved access to antibody testing and the identification of an expanding number of autoantibodies specifically associated with seizures. Autoimmune epilepsy has been linked to both neuronal cell surface antigens, such as LGI1, N-methyl-D-aspartate receptor (NMDA-R), and α-amino-3-hydroxy-5-methyl-4-isoxazolepropionic acid (AMPA), as well as neuronal intracellular antigens, including glutamic acid decarboxylase 65 (GAD65) [19]. While many of these autoantibodies cause seizures that typically respond well to immunotherapy, certain antibodies, such as GAD65, GABA(A)R, and LGI1, can lead to epilepsy. The diagnosis is frequently unclear even after a comprehensive evaluation, so empirical immunotherapy, which is considered the cornerstone of treatment, may be administered based on the clinician's judgment.

The sixth etiological category, designated as unknown, refers to patients whose etiology remains unclear [1]. An example of epilepsy of unknown cause is febrile infection-related epilepsy syndrome (FIRES), or epilepsy that occurs in school-age children after a febrile illness [20].

The main goal of this research was to determine the prevalence of etiological factors in adult patients in Herzegovina. Additional goals were to determine the prevalence of epilepsy concerning basic demographic characteristics (age, sex) and the type of epilepsy (focal, generalized, unknown).

This article was previously presented as a meeting abstract at the 2024 Mostar International Medical Congress MEDCORE, October 2024.

## Materials and methods

Research location and duration

The research was conducted at the Department of Neurology of the University Clinical Hospital Mostar from January 1, 2023, to December 31, 2023.

Research participants

The subjects were all patients over the age of 18 with a diagnosis of epilepsy of both sexes who were examined in the epilepsy outpatient clinic or hospitalized in the Department of Neurology in the period from January 2023 to December 2023. The exclusion criterion was all patients under the age of 18 of both sexes with a diagnosis of epilepsy in the specified period.

Data collection methods

With the approval of the Ethics Committee of the School of Medicine in Mostar (01-I-562/24), 421 patients were selected from the archives of the outpatient epilepsy clinic and the archives of the Department of Neurology of the University Clinical Hospital Mostar. After that, the following parameters were extracted from the hospital information system of the Department of Neurology in these patients: age, gender, type of epilepsy (focal, generalized, unidentified), and etiological factors of epilepsy (structural, genetic, metabolic, immunological, infectious, unknown). The emphasis during the search was on subtypes of structural factors, and they were singled out during data collection. The collected data were entered into a Microsoft Excel worksheet (Microsoft Corporation, Redmond, WA).

Study design

This was a retrospective study, in which patients' data were obtained from the archives of the outpatient epilepsy clinic and the archives of the Department of Neurology of the University Clinical Hospital Mostar.

Statistical analysis

Statistical analysis was done using IBM SPSS Statistics, version 25.0 (IBM Corp., Armonk, NY). The results are presented in tables and graphs. The chi-square test (χ2) was used to test for significant differences between parameters, and Fisher's exact test was used when the expected frequency was less than 5. The threshold for statistical significance was set at p < 0.05. P-values that could not be expressed to three decimal places were expressed as p < 0.001. Also, for statistical data processing, Student's t-test, one-way analysis of variance, and Scheffe's test were used.

## Results

**Figure 1:**
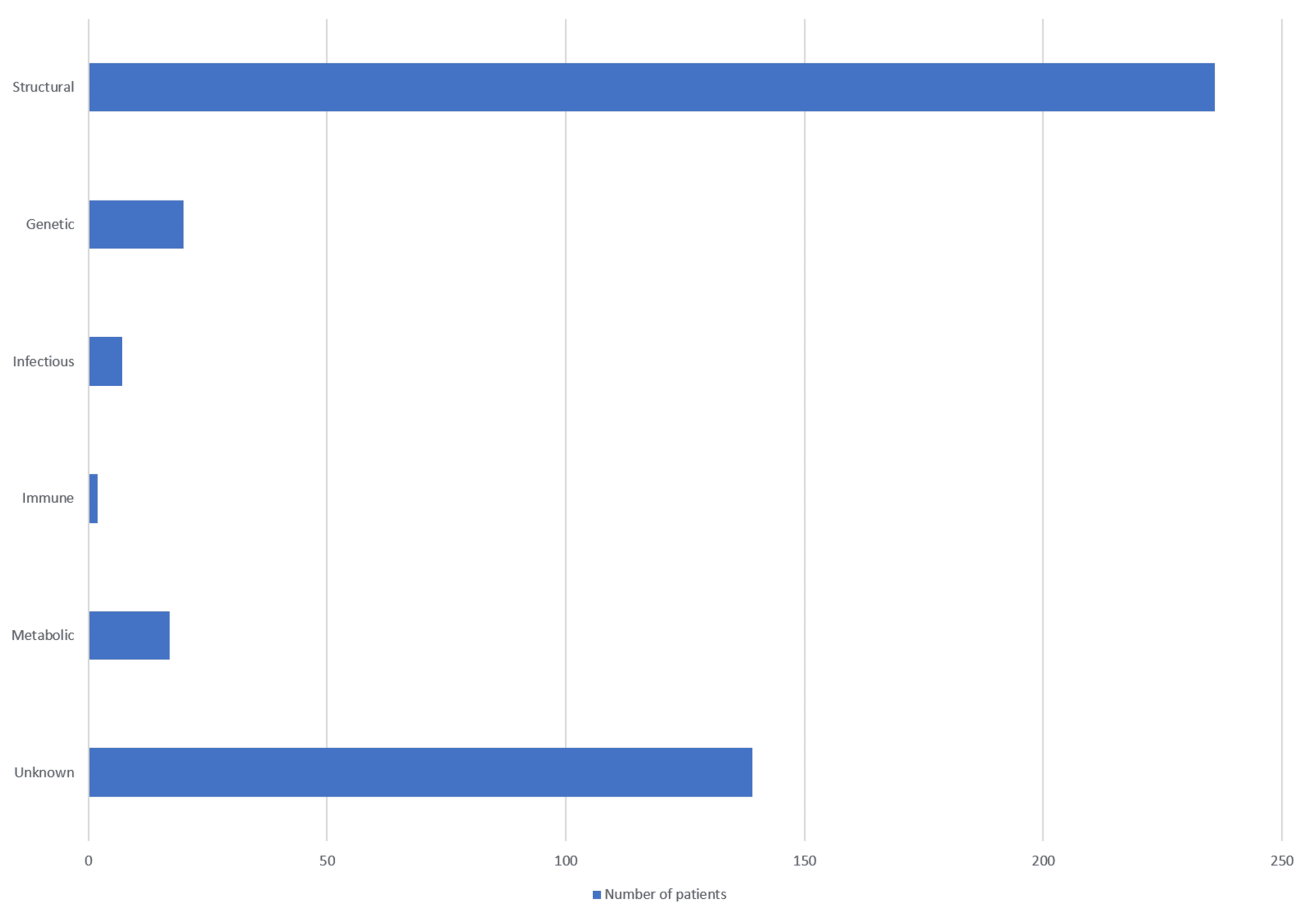
Etiology of epilepsy.

**Figure 2:**
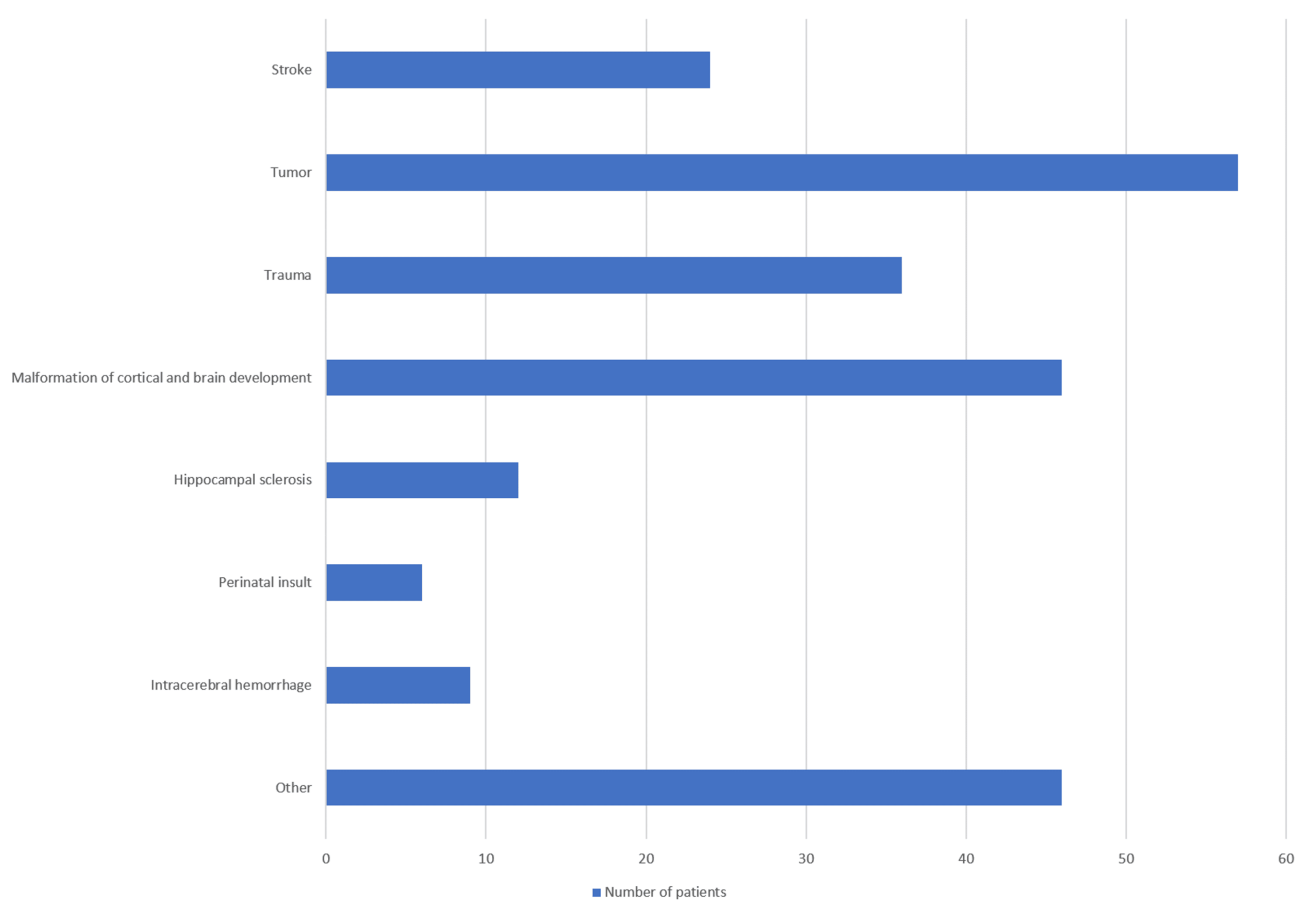
Etiology of structural epilepsy.

The study included 421 patients diagnosed with epilepsy who were treated in the epilepsy outpatient clinic or hospitalized at the Department of Neurology of the University Clinical Hospital Mostar from January 1, 2023, to December 31, 2023. All patients were over 18 years of age. Concerning the etiological factors of epilepsy in the studied sample, the most common etiological factors of epilepsy were structural, followed by unknown, genetic, metabolic, infectious, and the rarest immunological (Figure 1). Among the subtypes of structural etiological factors, the most common factors were tumors, followed by others, malformations of cortical and brain development, trauma, stroke, hippocampal sclerosis, intracerebral hemorrhage, and lastly, a perinatal insult (Figure 2).

No statistically significant difference was found between etiological factors and gender (Table 1).

**Table 1:** Association between gender and etiological diagnosis in patients with epilepsy. ** Fisher’s exact test.

	Gender				χ^2^	P
	Male		Female			
	N	%	N	%		
Unknown	61	29.9	78	35.9	1.474	0.225
Metabolic	7	3.4	10	4,6	0.134	0.715
Immune	1	0.5	1	0.5	0	1
Infectious	3	1.5	4	1.8	0	1**
Genetic	12	5.9	8	3.7	0.688	0.407
Structural	120	58.8	116	53.5	1.021	0.312

However, when we analyzed subgroups of structural epilepsies, the results showed that there was a statistically significant gender difference when it came to trauma as a cause of epilepsy, since brain trauma was a significantly more frequent cause in men than in women (Table 2).

**Table 2:** Association between gender and structural epilepsy. * Statistical significance (p < 0.05). ** Fisher’s exact test.

Structural	Gender				χ^2^	P
	Male		Female			
	N	%	N	%		
Stroke	16	7.8	8	3.7	2.650	0.104
Tumor	23	11.3	34	15.7	1.379	0.240
Trauma	26	12.7	10	4.6	7.892	0.005*
Malformation of cortical and brain development	19	9.3	27	12.4	0.761	0.383
Hippocampal sclerosis	4	2.0	8	3.7	0.594	0.441
Perinatal insult	4	2.0	2	0.9	0.238	0.437**
Intracerebral hemorrhage	7	3.4	2	0.9	2.080	0.097**
Other	21	10.3	25	11.5	0.061	0.805

When comparing structural epilepsy to other causes of epilepsy, a statistically significant difference was observed in the distribution of epilepsy types (focal, generalized, or unknown) (Table 3). Furthermore, within the structural epilepsy group, a significant difference was observed between patients with brain tumors and those with stroke, with focal epilepsy being more common in both cases (Table 4).

**Table 3:** Association between etiology and type of epilepsy. * Statistical significance (p < 0.05). ** Fisher’s exact test.

	Epilepsy						χ^2^	p
	Focal		Generalized		Unknown			
	n	%	n	%	n	%		
Unknown	49	23.7	87	44.2	3	17.6	21.056	<0.001*
Metabolic	4	1.9	12	6.1	1	5.9	4.661	0.097
Immune	1	0.5	1	0.5	0	0.0	0.086	0.958
Infectious	3	1.4	2	1.0	2	11.8	6.646	0.047**
Genetic	5	2.4	15	7.6	0	0.0	6.913	0.032*
Structural	145	70.0	80	40.6	11	64.7	36.051	<0.001*

**Table 4:** Association between structural epilepsy and epilepsy type. * Statistical significance (p < 0.05). ** Fisher’s exact test.

Structural	Epilepsy						χ^2^	
	Focal		Generalized		Unknown			
	N	%	n	%	N	%		
Stroke	16	7.7	4	2.0	4	23.5	16.573	<0,001*
Tumor	47	22.7	10	5.1	0	0.0	29.572	<0.001*
Trauma	19	9.2	15	7.6	2	11.8	0.550	0.760
Malformation of cortical and brain development	23	11.1	21	10.7	2	11.8	0.034	0.983
Hippocampal sclerosis	11	5.3	1	0.5	0	0.0	8.941	0.011*
Perinatal insult	3	1.4	3	1.5	0	0.0	0.261	1**
Intracerebral hemorrhage	5	2.4	3	1.5	1	5.9	2.237	0.360**
Other	21	10.1	23	11.7	2	11.8	0.256	0.880

Focal epilepsy was found to be the most common type of epilepsy, with a higher incidence in women than in men; however, without a statistically significant difference (Table 5).

**Table 5:** Association between gender and type of epilepsy. The threshold for statistical significance was set at p < 0.05.

	Gender				χ^2^	P
	Male		Female			
	n	%	n	%		
Focal	96	47.1	111	51.2	0.551	0.458
Generalized	98	48.0	99	45.6	0.159	0.690
Unknown	10	4.9	7	3.2	0.391	0.532

**Figure 3:**
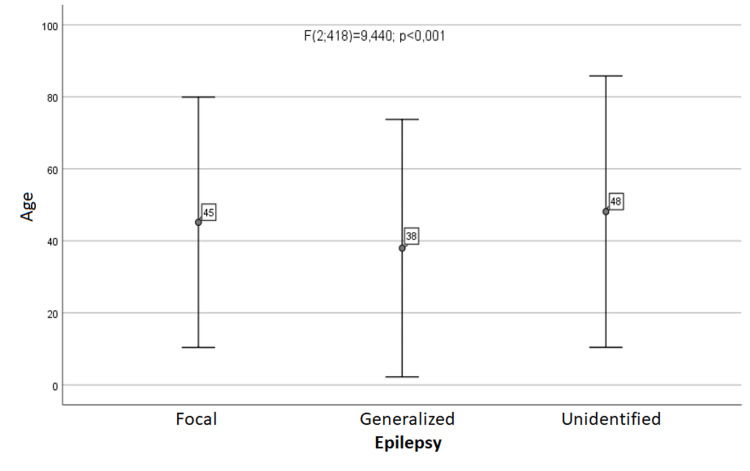
Association between age and type of epilepsy.

Using a one-way analysis of variance, significant age differences were found between the different types of epilepsy (Figure 3). Subsequent analysis using the Scheffe test showed that subjects with focal epilepsy were statistically significantly older than subjects with generalized epilepsy, while there were no statistically significant differences between the other groups.

## Discussion

Our study found that structural factors were the most common causes of epilepsy in patients evaluated at the Department of Neurology, University Clinical Hospital Mostar. Additionally, immunological causes were identified as the least common. Within the structural etiologies, tumors were the most prevalent, while perinatal insults were the rarest. In this study, we followed the classification adopted by the ILAE in 2017. The study conducted by Makkawi et al., which also used the same classification of epilepsy etiology, reported that the most common etiological factor was unknown and the rarest was metabolic, findings that differ from our study. Additionally, they identified stroke as the most common structural etiological factor, which also contrasts with our results [21]. On the other hand, Syvertsen et al. conducted a population-based study, from 1999 to 2014, which included 1771 patients diagnosed with epilepsy at Drammen Hospital and the National Center for Epilepsy at Oslo University Hospital. They followed the ILAE classification from 2010 and found that structural-metabolic was the most common etiological factor, which aligns with the findings of this study, while genetic causes were the least frequent. Stroke emerged as the most prevalent structural cause in their analysis, which may be explained by the fact that cerebral hemorrhage was not categorized separately as a distinct subtype, in contrast to our study. Approximately one-third of confirmed cases had an unknown etiology, highlighting the need for further investigation into the underlying causes of epilepsy [22]. Our results are consistent with the study performed by Buainain et al., which included 618 patients with epilepsy, with structural and unknown causes as the leading etiologies (41.1% and 38.5%, respectively), and immunological etiology being the rarest [23]. In a study conducted in Montenegro, the etiology of epilepsy was determined in just over a third of patients (34.4%). Among the symptomatic cases, stroke was the leading cause (30%), followed by primary brain tumors and traumatic brain injury (20% each) [24]. This is similar to our study, which also showed a high rate of unknown epilepsy; however, in our study, stroke ranked second among the etiologies. In a cross-sectional study conducted in Novi Sad, Serbia, involving 103 adolescents with active but uncomplicated epilepsy, the etiology of epilepsy was found to be unknown in 77.7% of the cases based on initial diagnosis and evaluation [25]. Our study found that the largest gender difference occurred in the group of patients where brain trauma was the cause of epilepsy, with trauma being more common in men. This can be attributed to the higher frequency of traffic accidents among men. In addition to the prevalence of etiological factors of epilepsy as the main goal of this study, it was determined that focal epilepsy was the most common type of epilepsy, which aligns with the numerous studies conducted to date [23,26-28]. Although most previous studies have reported a higher prevalence of epilepsy in men, our study found that epilepsy was slightly more common in women [21,23,26-28]. This discrepancy may be due to regional or population-specific differences in healthcare-seeking behavior, diagnostic practices, or access to medical services, where women may be more likely to seek medical attention and receive a diagnosis. Additionally, cultural or socioeconomic factors could influence reporting and detection rates differently across genders. It is also possible that hormonal influences or gender-related differences in underlying etiologies contribute to this variation, though further investigation would be needed to clarify these factors.

There are several strengths of this study that enhance its value and relevance. First, the findings have important clinical implications in everyday practice. Furthermore, it included data from both outpatient and inpatient settings, and followed patients for the entire calendar year. Moreover, the ILAE 2017 classification was used, which allows for comparability of the results with studies at the global level. Finally, taking into account the paucity of studies and the lack of previous data on the prevalence of etiological factors of epilepsy in Herzegovina, this study provides us with the first data on this topic and represents an important foundation for further research.

It is important to consider several key limitations when analyzing the results of our study. As a retrospective study, we relied on previous data, which may be incomplete and unreliable. This is especially true for cases with unclear etiology, where the absence of a history or diagnostic workup complicates the identification of the cause of epilepsy. Furthermore, the limited availability of advanced diagnostic tests, such as imaging or genetic tests, may also have played a role in this issue. Also, our results may not necessarily represent the broader population, as this was a single-center study. The predominance of tumors as the leading structural cause may be attributed to delayed diagnosis, environmental factors, or the possibility that more complicated cases were more frequently referred to our center. Despite these limitations, we believe that this study provides important insights and we expect it to be a good basis for future, larger multicenter studies with better diagnostic tools.

## Conclusions

Based on the findings of this study, the following conclusions can be drawn: structural factors were identified as the most common etiological cause of epilepsy, with immunological factors being the least frequent. Among structural factors, tumors were the most prevalent subtype, while perinatal insults were the rarest. Focal epilepsy emerged as the most common type, while cases of unknown epilepsy were the least frequent. Interestingly, epilepsy was found to be more prevalent in women than in men.

This study is the first to estimate the prevalence of epilepsy in Herzegovina, providing valuable insights into the epidemiology of epilepsy in this region. Furthermore, it highlights the significant issue of inadequate diagnostic capabilities, which may contribute to a higher rate of unknown etiologies. Given the importance of these findings, further research is essential, particularly multi-center studies with more comprehensive diagnostic tools, to gain a deeper understanding of epilepsy's etiology and improve clinical management in Herzegovina.
